# Unconventional Bearing Capacity Analysis and Optimization of Multicell Box Girders

**DOI:** 10.1155/2014/496398

**Published:** 2014-03-11

**Authors:** Jovan Tepic, Rade Doroslovacki, Mirko Djelosevic

**Affiliations:** ^1^Faculty of Technical Sciences Novi Sad, University of Novi Sad, 21000 Novi Sad, Serbia; ^2^Faculty of Mechanical and Civil Engineering Kraljevo, University of Kragujevac, 36000 Kraljevo, Serbia

## Abstract

This study deals with unconventional bearing capacity analysis and the procedure of optimizing a two-cell box girder. The generalized model which enables the local stress-strain analysis of multicell girders was developed based on the principle of cross-sectional decomposition. The applied methodology is verified using the experimental data (Djelosevic et al., 2012) for traditionally formed box girders. The qualitative and quantitative evaluation of results obtained for the two-cell box girder is realized based on comparative analysis using the finite element method (FEM) and the ANSYS v12 software. The deflection function obtained by analytical and numerical methods was found consistent provided that the maximum deviation does not exceed 4%. Multicell box girders are rationally designed support structures characterized by much lower susceptibility of their cross-sectional elements to buckling and higher specific capacity than traditionally formed box girders. The developed local stress model is applied for optimizing the cross section of a two-cell box carrier. The author points to the advantages of implementing the model of local stresses in the optimization process and concludes that the technological reserve of bearing capacity amounts to 20% at the same girder weight and constant load conditions.

## 1. Introduction

Typical box girders have rectangular or trapezoidal cross section and can be open or closed types. The open type is typically used in composite structures (such as bridges), whereas closed configurations are mainly used in steel structures. The traditional rectangular cross section is increasingly replaced by trapezoidal cross section which is more rational to use and the form of which is better suited for stresses in the girder [1]. Recognizing this fact, polygonal forms have been developed which are suitable from the aspect of local stress and loss of stability [2]. For highly stressed elements of the supporting structure to be designed rationally multicell box girders are required [3, 4]. These girder types are formed by integrating several forms of elements (cells) into a single functional unit which is characterized by reduced local stress and lower susceptibility to local loss of stability. The implementation of multicellular forms of various modifications to carrying bridge structures is manifested through high bearing capacity performances, especially in terms of stability and dynamic behaviour of the girder's elements (plates) [5]. The main reason for the application of multicellular box girders is reflected in their higher specific bearing capacity (capacity per unit of weight) compared to conventional solutions with rectangular cross section.

Traditionally designed box carriers are very susceptible to the local stress and loss of stability; thus, the impact of these phenomena is reduced by installing diaphragms and longitudinal stiffeners [6]. However, even despite these structural measures, the impacts of local stress and loss of stability are not completely eliminated; that is, their interaction has a significant share in the stress state of box girders. This especially applies to the pressed flange of girders whose buckling coefficient can be significantly reduced due to the effects of transverse load [7]. The phenomenon of stability of compressed plates is a subject of considerable number of studies; their behaviour is generally modelled by applying various numerical methods [8]. Studies of elastic stability of uniform vertical and longitudinally stiffened girder plates subjected to bending moment and live loads are presented in [9–12]. A significant contribution to this field is that of the analytical approach to stability analysis of perforated plates [13] which is based on a mathematical model, the basic assumptions of which are presented in [14–16]. The systematic analysis of local stresses in a transversely loaded box girder of rectangular cross section is provided in [17]. Developed is unconventional mathematical model of local stresses on the basis of which are identified influential parameters of carrying capacity and given guidelines for the optimal design of girders. It has been deemed consistent with the numerical method and the presented methodological approach was verified. The bearing capacity of vertical girder plates is mainly analyzed using numerical and experimental methods [18, 19]. The proper identification of stress state allows for defining the authoritative restrictions necessary in the process of optimization of elements of supporting structures. In the context of the above-cited studies especially important are the studies that analyze the biaxial compressed plates [20], box girders with diaphragms [21], and two-cell box girders [22].

## 2. Problem Definition and Model Assumptions

The bearing capacity of box girders is in functional dependence on geometric parameters of the girder and the active (external) load. Considering that in most cases the size and position of the load, as well as the girder length, cannot be influenced, it is clear that for given stress conditions the technological capacity reserve depends on the selection of appropriate (optimal) cross-sectional form. In general, the design of traditionally formed (rectangular) monocell box girders of large bearing capacity may be irrational due to the application of very thick plates. Namely, in order to meet the requirements regarding the box girder's bearing capacity, the flange plates must be at a sufficient distance from the neutral axis in order to ensure the necessary section modulus of the surface. This requirement calls for increasing the girder's height, while in order to prevent the lateral-torsional buckling it is also necessary to increase the girder's width proportionally. Increasing the girder's dimensions is exclusively related to the global bearing capacity condition.

The cross-sectional geometry which is defined by the global conditions is characterized by slender plates which are susceptible from the aspect of local stress. Local stresses cannot be reduced either by installing diaphragms or longitudinal stiffeners, or by increasing the thickness of compressed girder elements (plates).

**Figure 1:**
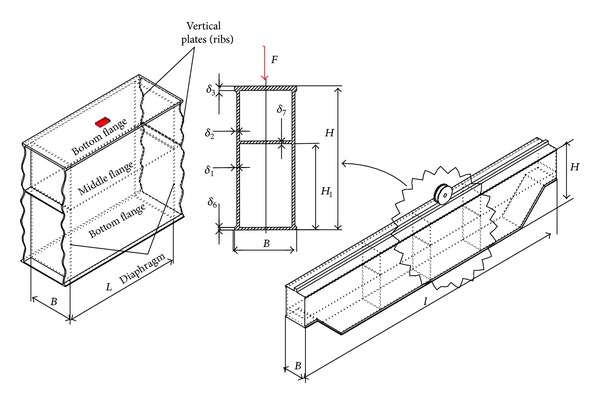
Structure and load of the two-cell box girders.

However, in case of very slender plates, installing stiffeners insufficiently effective and the local bearing capacity condition can be satisfied only by increasing their thickness. This reduces the rationality of mass and increases the structure's own weight, thus reducing the proportion of useful load in bearing capacity. The previously defined contradiction can be resolved by using multicellular girders. These constructive solutions are formed based on the analogy with the statically indeterminate line girders, and accordingly they are characterized by better capacity performances and lower susceptibility to local stresses. This is why in highly stressed and responsible support structures the use of multicell box girders is inevitable. The mathematical local stress model of the two-cell box girder is formed based on the following assumptions (Figure 1):

- length of the girder segment authoritative for the analysis is determined in accordance with the recommendations set forth in [17];
- the cross-sectional girder elements are considered as elastically restrained plates;
- displacements along the joint of girder elements are negligible compared to their deflection;
- the impact of plane forces is marginalized in relation to the active load and reaction moments of the elastic restrain;
- compressed girder plates are not susceptible to the loss of stability.

## 3. Mathematical Strain Model

**Figure 2:**
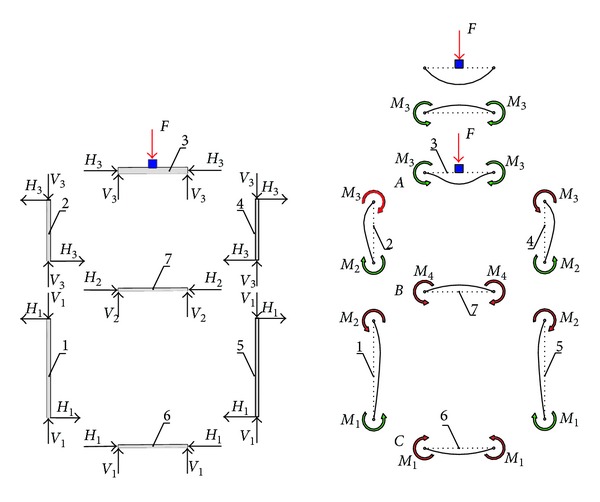
Internal reaction forces and moments of two-cell box girders.

Strain in the plate which is directly exposed to the external (active) load *F* on the other cross-sectional elements is manifested through the rotation joint zones (nodes). The slope on the side of panel through which the load is introduced into the monitored node has the same slope value as the corresponding changes of the other plates in the joint. This condition corresponds to the stress at which there is no relative change in the angle of the joint zone and no destruction of the cross-sectional elements; this condition should be satisfied during the girder's operation. The external load *F* acts symmetrically to the vertical girder axis. Thus, reactive torques in the elastic restrain can be determined based on the conditions defined for points A, B, and C (Figure 2). The impact of axial forces is negligible compared to the impact of moments through which the transverse forces manifested (in accordance with assumptions of the model). Values of moments of elastic restrain are determined using the principle of continuity of the girder's cross-sectional elements as follows.

 Node A
  (1) (∂w3(F)∂y3)y3=−(b3/2)+(∂w3(M1)∂y3)y3=−(b3/2) =(∂w2(M1,M2)∂y2)y2=b2/2.
 Node B
  (2) $$\begin{array}{c} {\left(\frac{\partial w_{1}\left(M_{2},M_{3}\right)}{\partial y_{1}}\right)}_{y_{1}=b_{1}/2}={\left(\frac{\partial w_{2}\left(M_{1},M_{2}\right)}{\partial y_{2}}\right)}_{y_{2}=-(b_{2}/2)}, \end{array}$$
  (3) $$\begin{array}{c} {\left(\frac{\partial w_{1}\left(M_{2},M_{3}\right)}{\partial y_{1}}\right)}_{y_{1}=b_{1}/2}={\left(\frac{\partial w_{7}\left(M_{4}\right)}{\partial y_{7}}\right)}_{y_{7}=-(b_{7}/2)}. \end{array}$$
 Node C
  (4) $$\begin{array}{c} {\left(\frac{\partial w_{1}\left(M_{2},M_{3}\right)}{\partial y_{1}}\right)}_{y_{1}=-(b_{1}/2)}={\left(\frac{\partial w_{6}\left(M_{3}\right)}{\partial y_{6}}\right)}_{y_{6}=b_{6}/2}. \end{array}$$

The first member, as defined by (1), is the slope at node A of the directly loaded simply supported plate (plate number 3) of the girder under the force *F*. Strain caused by this slope is the direct cause of stress in the plate of girder number 2, which has a tendency to reduce the pivoting of the edge of plate number 3 around the joint A zone. The physical interpretation of the preceding is reflected through the existence of a “torsion spring” whose stiffness depends on the strain of plate number 3, as well as the size of load which is transferred to plate number 2. The low stiffness of the torsion spring corresponds to the high slenderness of plate number 2, without affecting significantly the behaviour of plate number 3 which is considered as simply supported. Conversely, the higher thickness of plate number 2 allows for a significant share of load transferred through a more intense operation of reaction moments, so that the behaviour of plate number 3 corresponds to that of the fully restrained plate.

According to their characteristics, most of the finished girders correspond to the variant between the aforementioned extreme situations, while the mutual interactive relationship between the cross-sectional elements is regulated by the procedure of optimal design.

The second member of (2) defines the impact of plate number 2 through the moments of elastic restrain *M*_1_ operating along the edges of plate number 3. The third member of (1) represents the interaction effect of plate number 3, the moment of elastic restraint which has the same magnitude and opposite direction of plate number 2.

Deflection of the simply supported plate number 3 due to the impact of partial external load *F* is defined by the differential equation [14–16]

(5) $$\begin{array}{c} \frac{\partial^{4}w_{3}}{\partial x^{4}}+2\frac{\partial^{4}w_{3}}{\partial x^{2}y^{2}}+\frac{\partial^{4}w_{3}}{\partial y^{4}}=\frac{F}{uvD}. \end{array}$$

**Figure 3:**
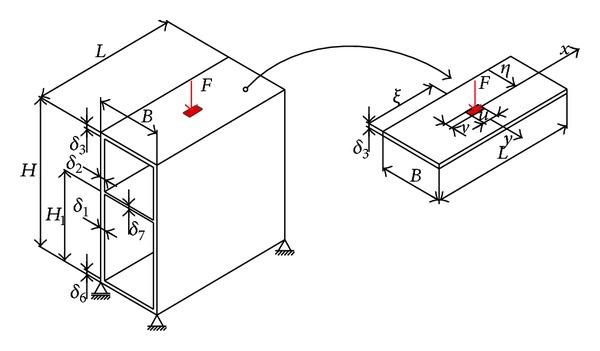
Box girder segment authoritative for the analysis of bearing capacity.

The simplest form of solution (5) is formulated through a double trigonometric series [14], which is characteristic only for use in simply supported plates (Figure 3); it is as follows:

(6) w3(F)=16FuvD3π6 ×∑m=1 ∞∑n=1∞((sin⁡mπξLsin⁡nπηB ×sin⁡mπu2Lsin⁡nπv2B)      ×(mn(m2L2+n2B2)2)−1) ×sinmπxLsinnπyB,

where *D*_3_ is flexural rigidity of plate number 3 and equals (*Eδ*_3_^3^/10.92).

The introduction of this term in order to investigate the bearing capacity of box girders is justified through numerical analysis and experimental verification [17].

Other girder plates are loaded only by reaction moments of the elastic restraint, whose role is to redistribute the active load to the individual cross-sectional elements. Functions of deflection for each *i*th plate of girder *w*_*i*_ (*i* = 1,…, 7) due to the operation of moments *M*_*i*_ and *M*_*i*+1_ along the edges of respective plates are defined by the following expression:

(7) wi(Mi,Mi+1) =L24π2Di∑m=1∞1m    2  ×[Mi,m+M(i+1),mcosh⁡αi,m    ·(αi,mtghαi,mcosh⁡mπyai−mπyaisinh⁡mπyai)    +M(i+1),m−Mi,msinh⁡αi,m     ·(αi,mctghαi,msinh⁡mπyai−mπyaicosh⁡mπyai)].

Reaction moments in the joint zones of cross-sectional elements *M*_1_, *M*_2_, *M*_3_, and *M*_4_ (Figure 2) are determined by the following series, respectively:

(8) $$\begin{array}{c} M_{1}=\overset{\infty }{\underset{m=1}{\sum }}E_{m}\sin\frac{m\pi x}{a}, \end{array}$$

(9) $$\begin{array}{c} M_{2}=\overset{\infty }{\underset{m=1}{\sum }}F_{m}\sin\frac{m\pi x}{a}, \end{array}$$

(10) $$\begin{array}{c} M_{3}=\overset{\infty }{\underset{m=1}{\sum }}G_{m}\sin\frac{m\pi x}{a}, \end{array}$$

(11) $$\begin{array}{c} M_{4}=\overset{\infty }{\underset{m=1}{\sum }}H_{m}\sin\frac{m\pi x}{a}, \end{array}$$

where *E*_*m*_, *F*_*m*_, *G*_*m*_, and *H*_*m*_ are coefficients of reaction moments as a function of parameter* m*.

Values of these coefficients are determined by substituting (6) and (7) into conditions of continuity (1)–(4), whereby the following equation system is derived:

(12) $$\begin{array}{c} E_{m}\cdot k_{1}+F_{m\cdot }\cdot k_{2}=S,\\ F_{m}\cdot k_{3}+G_{m\cdot }\cdot k_{4}=0,\\ E_{m}\cdot k_{5}+F_{m\cdot }\cdot k_{6}+G_{m}\cdot k_{7}=0,\\ F_{m}\cdot k_{8}+G_{m\cdot }\cdot k_{9}+H_{m}\cdot k_{10}=0, \end{array}$$

where *k*_*i*_ are cross-sectional geometric parameters (*i* = 1,…, 10); they are given in Appendix A. *S*_*m*_ is stress coefficient (load intensity and position); it is given in Appendix A.

Resolving the system (12) the required ratios of moments of elastic restraint *E*_*m*_, *F*_*m*_, *G*_*m*_, and *H*_*m*_ are obtained, which are given through the following formulations:

(13) $$\begin{array}{c} E_{m}=\frac{k_{3}k_{7}-k_{4}k_{6}}{\left(k_{3}k_{7}-k_{4}k_{6}\right)k_{1}+k_{2}k_{4}k_{5}}S_{m}, \end{array}$$

(14) $$\begin{array}{c} F_{m}=\frac{k_{4}k_{5}}{\left(k_{3}k_{7}-k_{4}k_{6}\right)k_{1}+k_{2}k_{4}k_{5}}S_{m}, \end{array}$$

(15) $$\begin{array}{c} G_{m}=-\frac{k_{3}k_{5}}{\left(k_{3}k_{7}-k_{4}k_{6}\right)k_{1}+k_{2}k_{4}k_{5}}S_{m}, \end{array}$$

(16) $$\begin{array}{c} H_{m}=-\frac{k_{3}k_{9}+k_{4}k_{8}}{\left(k_{3}k_{7}-k_{4}k_{6}\right)k_{1}+k_{2}k_{4}k_{5}}\frac{k_{5}}{k_{10}}S_{m}. \end{array}$$

Substituting (13)–(16) into (8)–(11) we obtain the distribution of moments of elastic restraint which operate in the joint zones of elements (plates) of the multicell girder. Defining the reaction moments, the causes of stress in individual cross-sectional elements were identified and the correlation dependence between the geometric parameters and the properties of active load is established. Based on the developed mathematical model, the following section contains the results of qualitative and quantitative analysis focusing on strains (deflections) of girder plates.

## 4. Analysis and Verification of Results

The methodology used for creating the mathematical model is based on the principle of decomposition of cross-sectional elements and was originally used in the analysis of bearing capacity of monocell box girders [17]. The principle of girder decomposition is featured by the possibility of its universal application, which allows for the use of more complex cross sections and multicell box girders. Studies dealing with traditional box girders of rectangular cross section have identified the zone of direct load effects (plates under the operation of active force) as a critical portion of girder from the aspect of local stress [17]. The width and thickness of this plate are the key parameters of bearing capacity, the value of which defines the degree of interaction with the vertical girder plates (ribs). Higher stress levels in the directly loaded girder plate (upper flange) are manifested through the proportional increase in the deflection of vertical plates (ribs) due to moments of elastic restraint. Since ribs of the box girder are generally loaded by biaxial pressure and tangential (shear) forces, these deflections all represent the initial values for curves of deflected surfaces and adversely affect the buckling phenomenon by reducing the critical stress values. Structural interventions aimed at reducing the deflection of vertical girder plates include the incorporation of stiffeners of appropriate form [23] or selecting the appropriate cross section [17]. The upper flange plate is uniaxially compressed, while the lower flange plate of the girder is subject to tension. Incorporating a midplate into traditional box girder forms two closed plate systems, creating thereby a two-cell configuration.

**Figure 4:**
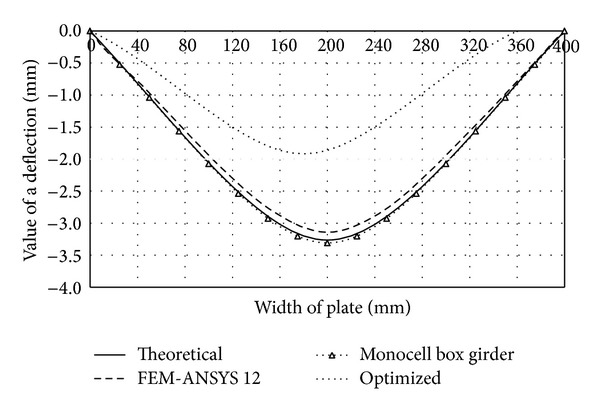
Comparative diagram of deflection of the girder's upper flange plate.

**Figure 5:**
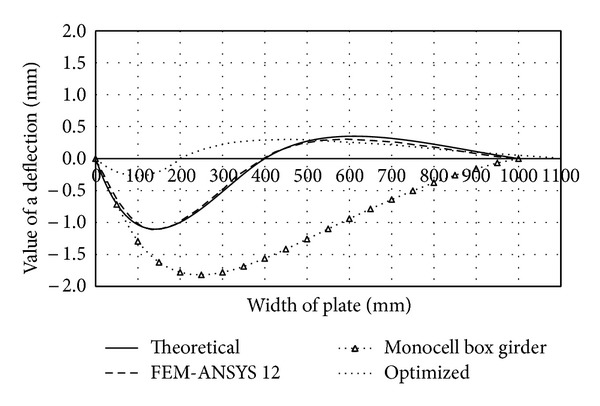
Comparative diagram of deflection of the left vertical girder plate (rib).

**Figure 6:**
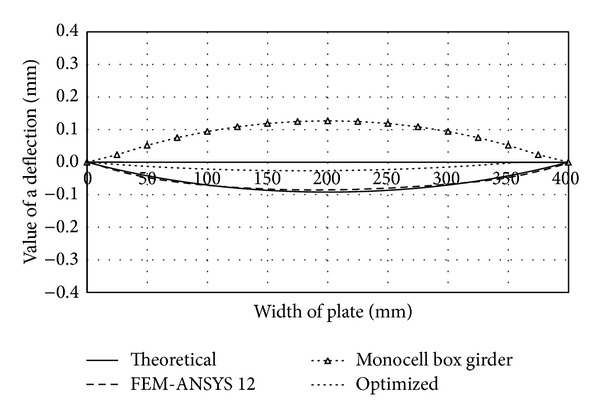
Comparative diagram of deflection of the girder's lower flange plate.

**Figure 7:**
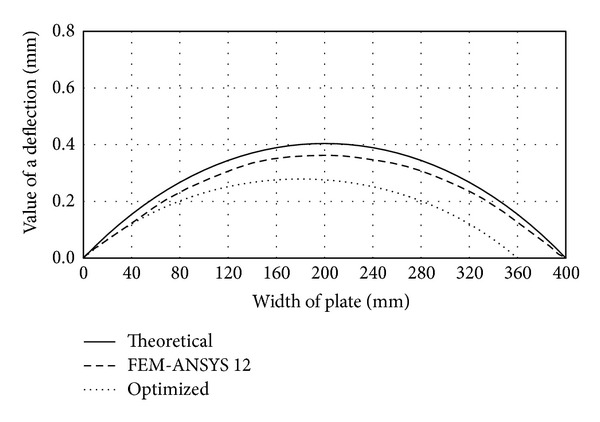
Comparative diagram of deflection of middle plate.

These constructive solutions are characterized by smaller cross-sectional width of the plate than in the case of traditional forms, which makes them more resistant to stress and less susceptible to loss of stability. By inserting several fields inside the box girder multicellular forms are created which are characterized by high specific bearing capacity and rational design. Performances of the monocell (traditional), two-cell (of same dimensions as the traditional form), and optimized two-cell girder are presented through the appropriate deflection functions and shown in comparative diagrams (Figures 4, 5, 6, and 7). Functions of deflection (Figures 4–7) were obtained by using (7) for the corresponding plates of multicell girder, while the initial and optimized dimensions of the girder are given in Table 2. Optimization is carried out according to the objective function (17) with the fulfillment of constraints (18)–(20). Results of optimization are the dimensions of the girder whose inclusion in (17) provides the maximum deformation with minimal use of materials for the making of the girder. Comparative analysis was performed according to the results obtained by applying the software ANSYS 12.

For generating model quadrangular finite elements of type SHELL 93 with 6 degrees of freedom per node were applied, which is the size of 10 mm.

The deflection function of the right vertical plate of the two-cell girder under consideration has the same form but opposite sign in comparison with the left element (Figure 5).

This study deals with the vertical two-cell box girder whose main role is to reduce the stress in vertical plates (ribs). By incorporating a midplate at height *H*_1_ we split the vertical plates into two parts and prevent the cross-sectional “opening" through the reduction of their deflection. The joint zone between the cross-sectional elements defines the inflection points of the corresponding deflection functions.

In the case of the two-cell girder under consideration, the inflection point of deflection function of the vertical plate (rib) is situated in the joint zone with the girder's midplate (point B). The physical interpretation of the inflection point refers to the change of sign of the deflection function and affects its shape, as well as its buckling mode. Namely, if the observed girder element (such as the vertical plate of Figure 5) contains an inflection point, the deflection function is characterized by the S shape.

By removing the midplate we obtain a monocellular (traditional) box girder, whose deflection function takes a C shape, regardless of the value of load and the cross-sectional geometric parameters. The C- or S-shaped deflection function is characterized by one and two half-waves, respectively, which are directly dependent on the buckling mode. The curve C corresponds to the first buckling mode and the critical force at which loss of stability occurs at substantially lower intensity than that in the S-shaped elastic surface. This is the main reason of stiffening the vertical plates and using multicell box girders. Comparative analysis (see Table 1) of deflection of the cross-sectional elements of the traditional and two-cell box girder for the same loading conditions (intensity and position of the force* F*) and the same cross-sectional area of the girder is graphically illustrated in Figures 4–7. In order to make the cross-sectional area of the considered girder types identical, the thickness of cross-sectional elements of the traditional box girder needs to be scaled up so that the overall change in their cross-sectional area corresponds to that of the midplate of the monocell girder.

Regardless of these measures, as indicated by the analysis of bearing capacity of the upper and lower flange plates, from the aspect of these flanges there is no essential difference between traditional and vertical two-cell box girders. Deflection of the upper flange plate of the monocell girder is higher by about 5%, while deflection of the bottom flange has opposite sign with an increase by about 38% as compared to the two-cell configuration. These values have great practical importance, because the difference for the upper flange is minor, while the bottom flange plate is the least loaded cross-sectional element, so the given increase of deflection does not affect the bearing capacity significantly. However, in case of vertical plates, a significant technological reserve of bearing capacity has been identified due to the S-shaped elastic surface.

**Figure 8:**
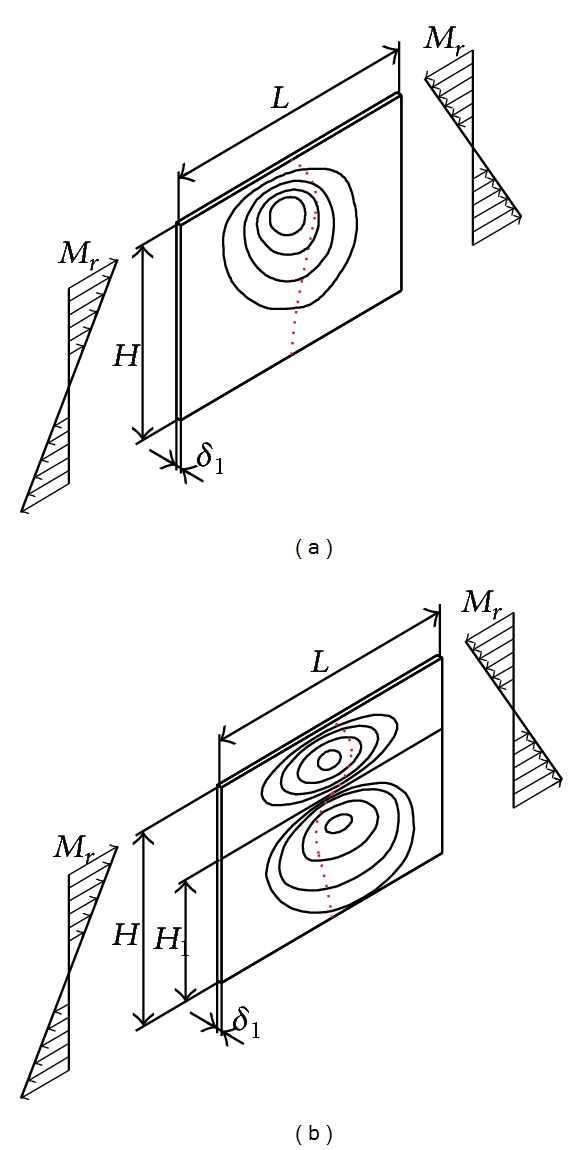
Buckling modes of the rib of the monocell (a) and two-cell (b) box girder.

Behaviour of the vertical plate of the monocellular girder depends solely on the relationship between the moments of elastic restraint initiated by the plates (marked red) and the reaction moments that oppose this strain (marked green, according to Figure 2). This interaction in the given cross section results in a concave curve of the elastic surface with the same sign. For the given load direction (Figure 3), part of the cross section above the neutral axis is compressed, while part of the girder beneath this axis is subject to tension. This suggests that the position of the midplate needs to be in the upper half of the girder's cross section (this is one of the structural-technological constraints in the optimization process). The largest share in the load acting upon the vertical girder plates is that of transverse reaction moments (8)–(11) and the global stress moment transferred by ribs *M*_*r*_ (acting in the plane of plate and is essential for the analysis of stability). Advantages of two-cell box girders over the traditional form are manifested through more uniform distribution of the two-wave deflection function (S-shaped) which corresponds to the higher value of critical stress (Figure 8).

## 5. Cross-Sectional Optimization of the Multicell Box Girder

The mathematical model developed in Section 3 has great theoretical and practical significance, not only for the capacity analysis but also for the procedure of cross-sectional optimization of two-cell box girders. The model can be successfully applied for the optimization according to stress or strain parameters, depending on the functional use of the box girder. Although the developed model relates to the girder's local behaviour, the compatibility of its application can be seen in the integration with the global conditions of capacity, which enables a more systematic approach to cross-sectional optimization.

Within this section, the author considers the optimization of a two-cell girder based on the criteria of local-global bearing capacity. Typical examples of the supporting structures to be optimized according to this criterion include crane rails and main girders of bridge cranes and the like. The goal function *f*_*goal*⁡_ involves minimizing the girder's weight (cross-sectional area); mathematically it can be expressed through the following expression:

(17) fgoal⁡=min⁡[B3δ3+B1δ1+2(H2−δ3)δ2    +2(H2−δ6)δ1+(B3−2δ1)δ7].

**Figure 9:**
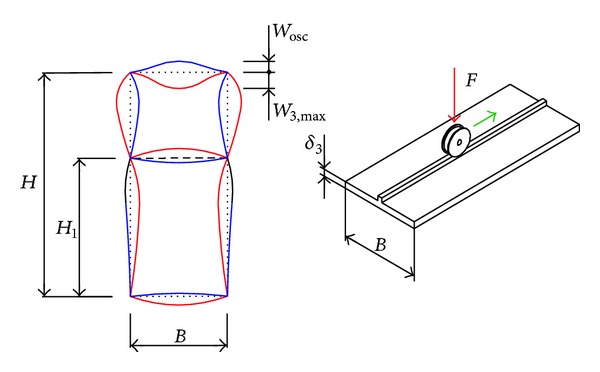
Oscillation of the girder elements due to the moving wheel.

Constraints that should be satisfied by the goal function (17) include the local and global conditions of bearing capacity. Local conditions are related to the limitations regarding the maximum deflection values of individual girder plates. Given that the deflection functions of girder elements define the local stress intensity, constraints regarding local conditions are indirectly related also to the local stress state. Practically, this means that in proportion to the reduction of the local plate deflections it simultaneously influences the reduction of local stresses in the corresponding cross-sectional elements. Through the deflection function of plates, the objective is to limit the intensity of local stress and strains and reduce the level of the girder elements' local oscillation *w*_osc_ (Figure 9).

Given that the most critical cross-sectional element is the directly loaded plate (Figure 3) and that the mathematical model establishes a functional dependence between the deflections of individual plates, it is fully justified for constraints regarding local conditions to be reduced only to this plate. In this case, constraints of local nature are mathematically formulated by (18), wherein local deflection *w*_3,max⁡_ is limited to the value of *w*_per_ = 2.0 mm. The *w*_per_ parameter has a dual role, that is, from the aspect of

- reducing the local dynamic influence manifested through local oscillations of cross-sectional elements as a result of changes in the position of the live load (wheel speed);
- indirect monitoring and managing the local stress intensity in order to eliminate potentially critical conditions (destruction of individual plate cross sections that could initiate the failure of girder).

The latter aspect implies that, by limiting the deflection of the directly loaded plate by force *F* to the value of *w*_per_, at the same time we reduce the maximum value of the von Mises stress *σ*_*e*_. Specifically, for the given geometric parameters according to the variant 1 (Table 2) and load conditions (Figure 3), the maximum value of deflection of the upper girder flange plate is *w*_3,max⁡_ = 3.26 mm and this deflection corresponds with the equivalent stress of *σ*_*e*,max⁡_ = 55.1 kN/cm^2^. Upon the optimization of the cross section, regardless of the fact that the thickness of the upper flange plate was not increased, the value of deflection is reduced by 75.2% and amounted to *w*_3,opt_ = 1.86 mm (adopted). This deflection value corresponds to the equivalent stress of *σ*_*e*,opt_ = 45.8 kN/cm^2^. The value *w*_per_ is selected so as to enable the reduction of voltage *σ*_*e*_ at least by 20%. Consider

(18) $$\begin{array}{c} w_{3,\max}=w_{3}\left(x=0,y=0\right)\leq w_{\text{per}}=2.0\;\left[\text{mm}\right]. \end{array}$$

Constraints that include global conditions are presented over the stress and strain criteria applicable to the linear girder model. Performances of the box girder of specific cross-sectional geometry and length *l* should allow for the transfer of load* F*, where maximum stress *σ*_max⁡_ and deflection *f*_max⁡_ values must be within the framework of permissible values (*σ*_per_ and *f*_per_). On the basis of these two constraints the global capacity conditions are defined for the case of a freely supported girder of length *l* (Figure 1).

Variables of the optimization procedure include all geometric parameters (dimensions) of the cross section (width and thickness of the girder plates). Constraints of local and global nature can be mathematically expressed by the following expressions:

(19) $$\begin{array}{c} \sigma_{\max}=\frac{Fl}{4W}\leq \sigma_{\text{per}}⟹W\geq \frac{Fl}{4\sigma_{\text{per}}}, \end{array}$$

(20) $$\begin{array}{c} f_{\max}=\frac{Fl^{3}}{4EI}\leq f_{\text{per}}⟹I\geq \frac{Fl^{3}}{48f_{\text{per}}}, \end{array}$$

where* E* is modulus of elasticity of the material (21000 kN/cm^2^),* W* is moment of resistance of the cross-sectional area (Appendix B), and* I* is axial moment of the cross-sectional area (Appendix B).

In addition to the global and local conditions, it is necessary for optimization procedure to include specific structural and technological solutions relating to plate thickness *δ*_*i*_ and the position of the midplate *H*_1_.

All plates should be thicker than 6 mm, while the position of the midplate should be in the top (compressed) part of the girder. The optimization process is implemented in Microsoft Excel using the Solver Module, while the results are shown in Table 2.

Studies dealing with the problem of cross-sectional optimization by using deterministic methods are solely based on the mathematical model of line girders, while constraints, in addition to global stress components, may include conditions of local bearing capacity and stability of cross-sectional elements. The quality of the optimization procedure depends largely on the number of constraints and the accuracy of their mathematical formulation, which is a factor that in many cases limits the proper implementation of this approach. This especially applies to the expressions defining the local stress and elastic stability, where mainly semianalytical and empirical expressions are used.

The above-presented optimization procedure is opposite to previous approaches, since the optimization procedure was based on the developed local stress model. The advantage of this concept is reflected through the explicit inclusion of local stress-strain conditions of bearing capacity in constraints of the goal function. On the other hand, due to the simplicity of formulation of the line model, the global characteristics of bearing capacity were integrated with the other constraints; thus, their influence was indirectly implemented in the goal function.

## 6. Conclusion

This study presented the analysis of bearing capacity and cross-sectional optimization of the two-cell box girder by applying an unconventional methodological approach. The generalized mathematical model of local stress was developed based on the principle of decomposition of the cross section. Implementation of the model is explained using the example of a vertical two-cell box girder while not diminishing the general nature of the presented methodology for the other multicell forms (with three or more cells). Performances of capacity of the two-cell box girder were evaluated in relation to the traditional form of the same dimensions and cross-sectional area. Based on the comparative analysis it was concluded that the midplate of the two-cell girder does not affect the capacity of the upper and lower flange plate to a significant degree, while predominantly influencing the vertical girder plates (ribs), reducing the values of deflection by 400%.

In addition, the deflection function of the vertical plate of the two-cell girder is S-shaped, which is favourable from the aspect of local stability of this cross-sectional element. The deflection function of vertical plates of traditional box girders always takes a C shape, which corresponds to the first buckling mode with considerable initial curvature. Susceptibility of the S-shaped deflection function to loss of stability is lower, since the initial deflection of the elastic surface corresponds to the second buckling mode which allows for higher values of critical forces. A specific significance of the presented research is related to the implementation of the mathematical model of local stress in the process of optimization of the two-cell box girder. The goal function represents the mass or cross-sectional area, while the optimization parameters (geometric variables of the cross section) include girder height* H*, girder width* B*, thickness of cross-sectional elements *δ*_*i*_ (*i* = 1,…, 7), and the position of the midplate *H*_1_. Constraints of the goal function are manifested through local, global, structural, and technological conditions.

Based on the implemented optimization significant technological reserve in bearing capacity has been achieved, especially in the upper flange and the vertical plates of the box girder, at the same girder mass and constant load conditions (the intensity and position of force were unchanged). The importance of this study has both theoretical and practical importance in the development and structural optimization of multicell girders. Through the presented methodology, the theoretical aspect enables the complete identification of local-global stress and allows a systematic approach to analyze the local stability of multicell box girders, which is important for further research work in this field. The practical implementation of results of this study enables the cross-sectional optimization of the multicell box girder beams, contributing to the development and design of the supporting structures of the high specific bearing capacity.

## Figures and Tables

**Table 1:** Comparative analysis of results of the two-cell box girder.

Plate of two-cell girder	Deformation and stress parameters of two-cell girder					
	Deflection (mm)		Reduction (%)	von Mises stress (kN/cm^2^)		Reduction (%)
	Figure 1	Optimized		Figure 1	Optimized	
1*	0.30	0.17	76.4	4.8	3.6	33.3
2*	1.10	0.27	400.0	13.2	12.1	9.1
3	3.26	1.86	75.3	55.1	45.8	20.0
6	0.09	0.03	300.0	3.6	2.5	44.0
7	0.40	0.28	42.8	7.8	6.4	21.8

*Values for plates 1 and 2 are identical to those for plates 4 and 5, respectively.

**Table 2:** Comparative analysis of influential box girder parameters.

Variant		The geometrical parameters of the cross section								Area	Deflection
Dimensions according to		*H*	*H*_1_	*B*	*δ*_1_	*δ*_2_	*δ*_3_	*δ*_6_	*δ*_7_	*A*	*w*_3_
		(mm)	(mm)	(mm)	(mm)	(mm)	(mm)	(mm)	(mm)	(cm^2^)	(mm)
1	Figure 1	1000	600	400	10	10	15	8	8	315	3.26
2	Optimized	1103	900	360	6	15	14.5	6	6	305	2.00
3	Adopted	1100	900	360	6	15	15	6	6	306	1.86

## Appendices

### A. Geometric Coefficients of Elastic Connection

(A.1) k1=D3D2(Bm,2+Cm,2)+2Bm,3,k2=D3D2(Bm,2−Cm,2),k3=D6D1(Cm,1−Bm,1),k4=−D6D1(Bm,1+Cm,1)−2Bm,6,k5=Bm,2−Cm,2,k6=D2D1(Bm,1+Cm,1)+Bm,2+Cm,2,k7=D2D1(Bm,1−Cm,1),k8=D7D1(Bm,1+Cm,1),k9=D7D1(Bm,1−Cm,1),k10=2Bm,7,Bm,i=−(αm,icosh⁡2αm,i+tghαm,i),Cm,i=(αm,isinh⁡2αm,i−ctghαm,i),αm,i=mπB2L,Sm=(4πD3a)(msin⁡(mπx/a))(∂w3∂x)|x=0=64Fuvπ4LB∑n=1∞((sin⁡mπξLsin⁡nπηB×sin⁡mπu2Lsin⁡nπv2B)      × ((m2L2+n2B2)2)−1).

### B. Polar and Axial Moment Cross Section

(B.1) $$\begin{array}{c} W\approx \frac{BH}{2}\left(\delta_{3}+\delta_{6}\right)+\frac{H^{2}}{6}\left(\delta_{1}+\delta_{2}\right),\\ I\approx \frac{BH^{2}}{4}\left(\delta_{3}+\delta_{6}\right)+\frac{H^{3}}{12}\left(\delta_{1}+\delta_{2}\right). \end{array}$$

## References

[1] Chidolue CA, Osadebe NN (2012). Flexural torsional behaviour of thin walled mono symmetric box girder structures. *International Journal of Engineering Sciences and Emerging Technologies*.

[2] Gonçalves R, Camotim D (2013). Buckling behaviour of thin-walled regular polygonal tubes subjected to bending or torsion. *Thin-Walled Structures*.

[3] Zhang X, Cheng G, Zhang H (2006). Theoretical prediction and numerical simulation of multi-cell square thin-walled structures. *Thin-Walled Structures*.

[4] Paolone A, Ruta G, Vidoli S (2008). Torsion in multi-cell thin-walled girders. *Acta Mechanica*.

[5] Zhang H, DesRoches R, Yang Z, Liu S (2010). Experimental and analytical studies on a streamlined steel box girder. *Journal of Constructional Steel Research*.

[6] Niezgodziński T, Kubiak T (2005). The problem of stability of web sheets in box-girders of overhead cranes. *Thin-Walled Structures*.

[7] Djelosevic M, Tanackov I, Kostelac M, Gajic V, Tepic J (2013). Modeling elastic stability of a pressed box girder flange. *Applied Mechanics and Materials*.

[8] John Wilson A, Rajasekaran S (2012). Elastic stability of all edges simply supported, stepped and stiffened rectangular plate under uniaxial loading. *Applied Mathematical Modelling*.

[9] Jaberzadeh E, Azhari M (2009). Elastic and inelastic local buckling of stiffened plates subjected to non-uniform compression using the Galerkin method. *Applied Mathematical Modelling*.

[10] Madhavan M, Davidson JS (2005). Buckling of centerline-stiffened plates subjected to uniaxial eccentric compression. *Thin-Walled Structures*.

[11] Ren T, Tong GS (2005). Elastic buckling of web plates in I-girders under patch and wheel loading. *Engineering Structures*.

[12] Maiorana E, Pellegrino C, Modena C (2008). Linear buckling analysis of unstiffened plates subjected to both patch load and bending moment. *Engineering Structures*.

[13] Djelosevic M, Tepic J, Tanackov I, Kostelac M (2013). Mathematical identification of influential parameters on the elastic buckling of variable geometry plate. *The Scientific World Journal*.

[14] Timoshenko SP, Woinowsky-Krieger S (1959). *Theory of Plates and Shells*.

[15] Timoshenko SP, Gere JM (1961). *Theory of Elastic Stability*.

[16] Allen HG, Bulson PS (1980). *Background to Buckling*.

[17] Djelosevic M, Gajic V, Petrovic D, Bizic M (2012). Identification of local stress parameters influencing the optimum design of box girders. *Engineering Structures*.

[18] Drdácý M (1991). On two particular problems of plate girder webs under partial edge loads. *Journal of Constructional Steel Research*.

[19] Granath P, Lagerqvist O (1999). Behaviour of girder webs subjected to patch loading. *Journal of Constructional Steel Research*.

[20] Farkas J, Simões LMC, Jármai K (2005). Minimum cost design of a welded stiffened square plate loaded by biaxial compression. *Structural and Multidisciplinary Optimization*.

[21] Megson THG, Hallak G (1995). Optimum design of load-bearing box girder diaphragms having a central support. *Thin-Walled Structures*.

[22] Cardoso JB, Valido AJ (2011). Cross-section optimal design of composite laminated thin-walled beams. *Computers and Structures*.

[23] Graciano C, Johansson B (2003). Resistance of longitudinally stiffened I-girders subjected to concentrated loads. *Journal of Constructional Steel Research*.

